# A Systematic Review of WNT Signaling in Endothelial Cell Oligodendrocyte Interactions: Potential Relevance to Cerebral Small Vessel Disease

**DOI:** 10.3390/cells9061545

**Published:** 2020-06-25

**Authors:** Narek Manukjan, Zubair Ahmed, Daniel Fulton, W. Matthijs Blankesteijn, Sébastien Foulquier

**Affiliations:** 1 Department of Pharmacology and Toxicology, Maastricht University, PO Box 616, 6200 MD Maastricht, The Netherlands; n.manukjan@maastrichtuniversity.nl or wm.blankesteijn@maastrichtuniversity.nl (W.M.B.); 2 CARIM—School for Cardiovascular Diseases, Maastricht University Medical Center+, PO Box 616, 6200 MD Maastricht, The Netherlands; 3 Neuroscience and Ophthalmology, Institute of Inflammation and Ageing, University of Birmingham, Birmingham B15 2TT, UK; z.ahmed.1@bham.ac.uk (Z.A.); d.fulton@bham.ac.uk (D.F.); 4 Department of Neurology, MHeNs—School for Mental Health and Neuroscience, Maastricht University Medical Center+, PO Box 616, 6200 MD Maastricht, The Netherlands

**Keywords:** cerebral small vessel disease, WNT, β-catenin, oligodendrocytes, oligodendrocyte precursor cells, endothelial cells

## Abstract

Key pathological features of cerebral small vessel disease (cSVD) include impairment of the blood brain barrier (BBB) and the progression of white matter lesions (WMLs) amongst other structural lesions, leading to the clinical manifestations of cSVD. The function of endothelial cells (ECs) is of major importance to maintain a proper BBB. ECs interact with several cell types to provide structural and functional support to the brain. Oligodendrocytes (OLs) myelinate axons in the central nervous system and are crucial in sustaining the integrity of white matter. The interplay between ECs and OLs and their precursor cells (OPCs) has received limited attention yet seems of relevance for the study of BBB dysfunction and white matter injury in cSVD. Emerging evidence shows a crosstalk between ECs and OPCs/OLs, mediated by signaling through the Wingless and Int-1 (WNT)/β-catenin pathway. As the latter is involved in EC function (e.g., angiogenesis) and oligodendrogenesis, we reviewed the role of WNT/β-catenin signaling for both cell types and performed a systematic search to identify studies describing a WNT-mediated interplay between ECs and OPCs/OLs. Dysregulation of this interaction may limit remyelination of WMLs and render the BBB leaky, thereby initiating a vicious neuroinflammatory cycle. A better understanding of the role of this signaling pathway in EC–OL crosstalk is essential in understanding cSVD development.

## 1. Introduction

Cerebral small vessel disease (cSVD) is an umbrella term used to describe different pathological processes that affect the small vessels of the brain, including small arteries, arterioles, capillaries, and small veins, and is associated with brain structural lesions and eventual cognitive impairment [1]. With cSVD accounting for up to 25% of all stroke and 45% of dementia, it is seen as a common cause of cognitive impairment worldwide [2]. Beyond cognitive impairment, mood and gait disorders are also often seen in cSVD patients [3]. Although recent advances have led to an improved diagnosis and understanding of cSVD, its exact pathogenesis is still unknown. However, damage to the blood brain barrier (BBB) is recognized as an early pathological step in cSVD, often resulting from the exposure to cardiovascular risk factors, such as hypertension and diabetes [2,4,5].

BBB dysfunction has been evidenced in experimental animal models of cSVD as well as in cSVD patients [6,7,8]. Endothelial cells (ECs) are essential for BBB function due to their tight junction (TJ) proteins that prevent the passive entrance of cells and macromolecules into the brain. Endothelial dysfunction is therefore a key contributor to BBB dysfunction and is present in several brain pathologies, including stroke, multiple sclerosis (MS), and Alzheimer’s disease [9]. In addition to the endothelial barrier, BBB integrity also relies on non-neuronal cells, such as pericytes, astrocytes, and oligodendrocytes (OLs) [10]. Although studies are scarce, the involvement of different cell types, especially OLs, might play a critical role in the pathophysiology of cSVD. Both OLs and oligodendrocyte precursor cells (OPCs) are able to lower the brain endothelial permeability when co-cultured with primary brain ECs [11]. In addition, OL dysfunction can cause white matter loss and BBB disruption, two important features seen in cSVD patients [10,12,13]. A common factor that may facilitate an interaction between ECs and OPCs/OLs is platelet-derived growth factor (PDGF) as ECs and OPCs secrete PDGF and express its receptor (PDGFRα). The tightening of the BBB by OLs seems, however, not to depend on the PDGF/PDGFRα signaling, as it cannot be prevented by a PDGFRα antagonist [11]. This suggests that other pathways might play a critical role in the interaction between ECs and OPCs/OLs. The Wingless and Int-1 (WNT)/β-catenin pathway, largely described for its function within ECs and OPCs/OLs may be crucial in this crosstalk [14,15].

Although both ECs and OLs (and their precursor cells) are involved in BBB integrity, studies examining these cells and their interaction in the context of cSVD are limited. This review aimed to summarize the published findings on the crosstalk between ECs and OPCs/OLs, and in particular the role played by WNT/β-catenin signaling in this interaction. First, we briefly address the respective roles of WNTs in ECs and OPCs/OLs. Second, we discuss the current knowledge on EC–OL interaction. Third, we present studies found via a systematic search that have identified a role for WNT signaling in this crosstalk. Finally, we discuss the potential implications of these findings for cSVD pathology before we formulate a conclusion.

## 2. Pathology of Cerebral Small Vessel Disease

cSVD is a heterogeneous disease that includes genetic and sporadic forms. Sporadic cSVD, the most prevalent form of cSVD, can be further subdivided into amyloidal and non-amyloidal subtypes [1,10,16]. Sporadic cSVD is associated with age and the presence of cardiovascular risk factors. Hypertension is considered as the major modifiable risk factor for the development of cSVD and vascular cognitive impairment [16,17]. The thickening, stiffening, and narrowing of extra-cranial and cranial vessels induced by hypertension can lead to brain hypoperfusion, with subsequent hypoxia, BBB impairment, neuroinflammation, and the appearance of white matter lesions (WMLs), ultimately leading to demyelination and neurodegeneration [18,19,20,21]. The pathological cascade induced by hypertension has been summarized by the ‘three hit’ hypothesis [22]. The initial hit is hypertension itself, which can initiate an ischemic insult leading to local tissue hypoxia. The local hypoxic state, which is considered as the second hit, can promote damage and inflammation, which represents the third and final hit. In the course of this pathological cascade, hypoxia can increase BBB permeability and induce myelin breakdown either directly and/or indirectly via the expression of cytokines, such as tumor necrosis factor (TNF)-α and interleukin (IL)-1β, secreted by microglia and macrophages. Reactive microglia and brain macrophages can alter the BBB integrity itself and also damage the white matter tissue and its supporting cells, such as OLs, leading to myelin breakdown and vasogenic cerebral edema [22]. Altogether, neuroinflammation and myelin breakdown can cause WMLs and the development of cognitive impairment [22,23].

Taken together, our current knowledge on cSVD pathology indicates a role for ECs and OLs in maintaining the integrity of the white matter and the BBB, and suggests that their dysfunction could be critical in the course of cSVD development.

## 3. Brain Endothelial Cells

Brain ECs harbor specific features compared to peripheral ECs, namely the presence of TJ proteins. This is a key feature in maintaining the central nervous system (CNS) microenvironment and to protect the brain from invading pathogens, immune cells, toxic compounds, or alternating ionic concentrations that can affect normal cerebral function [24]. Pericyte and astrocyte end-feet support this barrier by surrounding the EC [25,26]. TJ link adjacent ECs together by forming homodimer transmembrane proteins, which are important in maintaining the tightly regulated brain microenvironment. Dysfunction of these proteins can cause leakage of the BBB and disruption of brain functions [27]. The brain requires certain nutrients to maintain its normal function, thus controlled permeability is required for normal brain function. The EC in the BBB are specialized in allowing the transport of certain molecules and cells when needed, such as glucose for energy or immune cells during inflammation [28,29]. 

ECs also play a key role in regulating local cerebral blood flow in response to neuronal activity and their underlying metabolic needs. This is regulated by the relaxation of smooth muscle cells and pericytes surrounding the larger vessels and capillaries, respectively, mediated by the release of nitric oxide (NO), synthesized by the endothelial NO synthase (eNOS). Upon eNOS deactivation, or impaired vasodilation, the cerebrovascular tone is increased, potentially leading to hypoxia in the corresponding areas [24]. This highlights the importance of tight regulation of the vascular diameter by ECs and the adverse consequences of malfunctioning of this system. 

Microvascular dysfunction has been proposed as an early sign of cSVD, preceding the occurrence of structural lesions, such as WMLs. Endothelial dysfunction causes BBB leakage and inflammation, which has been suggested to cause lacunar strokes, white matter hyperintensities, and cerebral microbleeds seen in cSVD [2,24,27,30]. An increased plasma concentration of markers of endothelial activation, such as vascular cell adhesion molecule (VCAM)-1 and intracellular adhesion molecule (ICAM)-1, has been observed in patients with cSVD compared to control subjects [30]. Furthermore, beyond their own dysfunction, the release of detrimental mediators by diseased ECs may also affect other brain cells, further aggravating cSVD progression [24].

## 4. Oligodendrocytes

OLs are the cells responsible for the production of myelin, the isolating fatty sheath surrounding axons that provides structural protection, and facilitate fast electrical signal transduction along CNS axons. In addition to this role in action potential conduction, recent work has revealed additional roles in various processes, including trophic and metabolic support of neurons [31,32,33,34,35], information processing in neural circuits [36], and interactions with other CNS cell types, such as ECs [37,38]. 

Myelinating OLs are generated through a sequence of developmental steps involving four stages: OPCs, late OPCs, immature OLs, and mature myelinating OLs [39]. During this developmental sequence, OPCs migrate to sites requiring myelination, where they proliferate and undergo a morphological differentiation that marks their transition into late OPCs (also known as preoligodendrocytes). Late OPCs exhibit highly branched process arbors whose terminal branches make contact with numerous neuronal compartments, including axons [40,41]. This developmental sequence continues as late OPCs exit the cell cycle and differentiate into immature OLs that upregulate the expression of myelin genes and convert some of their initial axonal contacts into loose membrane wraps. As OL maturation progresses, these early ensheathments are elongated and compacted to form mature myelin sheaths. Other non-myelinating process branches are resorbed so that in the final mature OL, all process branches support myelinating segments. Of note, a significant number of OPCs persist in CNS tissues beyond the period of developmental myelination, where they provide a pool of precursors that can be recruited in the case of injury [42,43,44]. The rate of OPC differentiation decreases in the adult CNS [43], and while some OPCs differentiate to produce myelinating OLs, the majority remain as OPCs and are not involved in myelin sheath production [42]. Yeung et al. suggest that human OLs have the ability to remodel the myelin sheaths without the need for the generation of OLs from OPCs [45]. However, other studies show that there is a gradual production of newly differentiated OLs that engage in de novo myelination and myelin remodeling in the adult CNS [46,47]. Factors released by OPCs and OLs, such as insulin-like growth factor 1 (IGF-1) and brain-derived neurotrophic factor (BDNF), induce neuronal cell survival [31,48], indicating that OLs indeed provide important trophic support for axonal maintenance and survival. Hypertension, hypoxia, and inflammation might disrupt these factors, leading to OL cell death and damage to the integrity of white matter tissues. Loss of white matter due to hypoperfusion-induced OL death and myelin loss in cSVD has been demonstrated in experimental animal models [49,50,51]. Cognitive impairment associated with the loss of OLs has been observed, for instance, following brain hypoperfusion in rats via the permanent bilateral occlusion of the common carotid arteries [49]. Hypoperfusion led to an initial increase in OPCs, which was followed by an increase in OPC cell death that produced a net decrease in the number of mature OLs in this chronic hypoperfusion rat model [50]. A similar finding was observed in spontaneously hypertensive rat-stroke prone (SHRSP) subjected to brain hypoperfusion in association with increased inflammatory mediators [51]. Overall, the complex multi-step process involved in OL development and function, and its dependence on tightly controlled regulatory mechanisms, renders OLs highly sensitive to pathological conditions in both the developing and mature CNS. 

Consequently, the emergence of a local or global brain hypoperfusion can affect both OPC/OL function and the factors secreted by these cells under these conditions. One group of these secreted factors are the WNT proteins and their signaling molecules, an upregulation of which was found after exposure of OPCs to hypoxic conditions, suggesting that they may play major roles in brain hypoxia [52]. In fact, these proteins play a complex role in both white matter integrity and angiogenesis, with a key role in both OPC fate, OL survival, and EC proliferation [14,15,52]. Taken together, these findings suggest that WNT signaling may be a key regulator of OPC–OL interaction, and an important mediator in cSVD pathology.

## 5. WNT Signaling in Endothelial Cells and Oligodendrocytes

### 5.1. WNT Signaling

**Figure 1:**
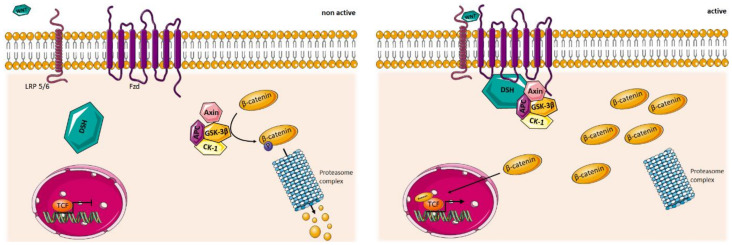
Schematic representation of the Wingless and Int-1 (WNT)/β-catenin signaling pathway. In the non-active state (**left panel**), β-catenin is phosphorylated by a multi-component complex containing glycogen synthase kinase 3β (GSK-3β), Axin, adenomatous polyposis coli (APC), and casein kinase-1 (CK-1). This causes β-catenin to be phosphorylated for degradation by the proteasome complex. In the active state (**right panel**), a WNT ligand binds to its receptor and co-receptor, Frizzled (Fzd), and low density lipoprotein (LDL) receptor-related protein 5 or 6 (LRP5/6), respectively, and causes the recruitment of components of the β-catenin phosphorylating complex to the membrane together with disheveled (DSH). This leads to the cytoplasmic accumulation of β-catenin and its translocation to the nucleus, eventually binding members of the T-cell factor/lymphoid enhancing factor (TCF/LEF) transcription factors and activating transcription of target genes.

WNT signaling is typically categorized into two pathways: The β-catenin- and non-β-catenin-mediated pathways, with the latter being further sub-categorized. The non-beta-catenin-mediated pathways involve all WNT pathways that do not lead to the stabilization of β-catenin and plays a role in processes including cell polarization, cell fate, inflammatory response, and cell migration [53]. The β-catenin-mediated WNT signaling pathway leads to the intracellular stabilization of β-catenin, resulting in its translocation to the nucleus and the transcription of numerous genes involved in cell proliferation, differentiation, tissue expansion, cell fate, and many more [54]. In the absence of WNT proteins, β-catenin is phosphorylated by a protein complex formed by glycogen synthase kinase 3β (GSK-3β), Axin, adenomatous polyposis coli (APC), and casein kinase-1 (CK-1). Phosphorylation of β-catenin by this so-called destruction complex leads to its degradation by the ubiquitin proteasome. Secreted WNT proteins require two distinct receptor families for their intracellular signaling, namely the Frizzled (Fzd) and the low-density lipoprotein (LDL) receptor-related protein 5 or 6 (LRP5/6). Binding of WNT to Fzd and its co-receptor LRP5/6 results in the recruitment of the disheveled (Dsh) protein to the plasma membrane. This leads to the recruitment of several components of the β-catenin phosphorylation complex and their inhibition, which in turn leads to the accumulation of β-catenin in the cytoplasm. Increased cytoplasmic β-catenin promotes its translocation to the nucleus and its binding to transcription factors, leading to transcription of its target genes (Figure 1) [14]. An extensive description of these different WNT signaling pathways is beyond the scope of this review but can be found in a recent publication from our group [14].

### 5.2. WNT Signaling in Brain Endothelial Cells

β-catenin is essential for the formation and maintenance of vascular integrity and controls BBB TJ formation [55,56]. The first steps of blood vessel formation seem to rely on WNT/β-catenin signaling promoting EC-specific differentiation of pluripotent stem cells, an essential step in vasculogenesis (the initial blood vessel formation) [14]. WNT/β-catenin also induces the expression of TJ and glucose transporter 1 (Glut1) proteins, which are key features of brain ECs. Besides differentiation, several WNTs have also been reported to induce EC proliferation and migration to promote vessel assembly [57]. WNT signaling is also essential for the formation of new blood vessels from pre-existing ones, called angiogenesis [14]. In a non-angiogenic state, cytoplasmic β-catenin is constantly degraded and thus does not result in the formation of new vasculature. When WNT signaling is activated, stabilization of β-catenin leads to angiogenesis. Angiogenesis is further regulated by the negative feedback mechanism involving the activation of c-Casitas B-lineage lymphoma (c-Cbl). Phosphorylated c-Cbl promotes the degradation of active β-catenin, and thus functions as a negative regulator of angiogenesis [58]. CNS angiogenesis has been shown to be dependent dominantly on the β-catenin-mediated WNT signaling ligands WNT7a and WNT7b [59]. These ligands are dependent on coactivators G protein-coupled receptor 124 (GPR124) and Reck expressed by the EC, which enhance WNT7a/7b signaling pathways [60]. Benz et al. showed that brain areas lacking a BBB have ECs with low levels of β-catenin activation due to the absence of WNT receptors. Increased β-catenin activation resulted in increased endothelial expression of the TJ protein claudin5, and was associated with a reduction of BBB permeability in the corresponding areas [61]. In addition to angiogenesis, the expression of TJ proteins, such as claudin3 and claudin5, which maintain BBB integrity, is also regulated by WNT/β-catenin signaling [56,61]. In vitro stabilization of β-catenin with WNT3a treatment in primary ECs resulted in an increased expression of claudin3, and the formation of TJ and BBB characteristics [56], while in vivo overactivation of β-catenin in transgenic mice led to an increased expression of claudin5 [61].

Taken together, this demonstrates the essential role of WNT/β-catenin signaling in the formation and integrity of the BBB. Interestingly, WNT7a and WNT7b, which drive angiogenesis, are the predominant WNT proteins expressed by OPCs [52,62].

### 5.3. WNT Signaling in Oligodendrocytes

WNT/β-catenin signaling was initially suggested to exert an inhibitory effect on oligodendrogenesis and differentiation, although it is now clear that this pathway regulates multiple events during the OPC developmental stages [15,63,64]. While some studies have identified a WNT/β-catenin-mediated repressive function for the OPC specification from neuronal stem cells (NSCs) during prenatal development [63,65], other studies showed that WNT signaling was dispensable or could even enhance OPC differentiation [66,67]. It has become clear that WNT signaling plays a complex role in the fate of OPCs in a context-dependent manner, depending on the developmental stage, location in the CNS, cell type, exposure level, and possible interactions with other pathways affecting the cell fate [15,64]. Guo et al. proposed a working model on the multimodal role of WNT/β-catenin signaling in OPC development [15]. Low levels of β-catenin signaling promote OPC differentiation to immature OLs during development, whereas high levels inhibit OPC density and differentiation. Similar findings were observed during OL maturation, where the exact role of WNT/β-catenin signaling on OL maturation remains unclear [15]. Olig2Cre; Da-Cat mice, which have dominant-active β-catenin in OPCs and OLs, had decreased numbers of myelin proteolipid protein (PLP) expressing OLs, which was associated with hypomyelination, while OPC numbers were not affected [66]. On the contrary, Tawk et al. reported that WNT/β-catenin signaling plays a role in activating this myelin gene in OLs. They showed that inhibition of WNT signaling components by small interfering RNA (siRNA) resulted in a decrease in *plp* expression levels, while a three-fold increase in the expression of this myelin gene protein was observed following WNT/β-catenin activation by WNT1 [67]. Myelin is a major component within white matter, and damage to myelin proteins is widely seen in cSVD as discussed previously. It is therefore important to clarify the effects of WNT signaling on OPC differentiation and maturation in the context of demyelination and remyelination. 

After demyelination, activation of WNT/β-catenin seems to inhibit OPC differentiation and to decrease myelin density. Fancy et al. reported a decrease in PLP-expressing OLs, 14 days after a lysolecithin (LPC)-induced lesion in Olig2Cre;Da-Cat mice. They also found a decrease in remyelination after LPC-induced demyelination in mice lacking one *Apc* allele or containing a complete deletion of the *Axin2* gene, which both caused an increase in β-catenin levels [66]. In line with this finding, the inhibition of WNT/β-catenin by aspirin was associated with increased OL differentiation [68]. However, in another study, neither *Apc* single-allele conditional knockout nor one-allele nonsense truncated mutation differed in the levels of WNT/β-catenin signal when compared to wild type (WT) mice [69]. This suggests that the previous reported delay in OPC differentiation might have resulted from β-catenin-independent effects of *Apc* deletion. On the contrary, an increase in the WNT transcription mediator gene, transcription factor-4 (TCF4 (also known as TCF7L2)), was reported following white matter demyelination in rodents and in active areas of MS lesions, indicating a beneficial role for WNT signaling in remyelination [66]. It was, however, demonstrated that TCF4 was expressed only early in remyelination in both mice and MS patients, and not in later stages or in chronic lesions [70,71]. This might indicate an early OPC recruitment via WNT/β-catenin signaling, which has been reported to play a role in OPC migration and the attachment to vessels [72], as a rescue mechanism in response to demyelination.

Although sometimes contradictory at first sight, these results highlight a crucial role for WNT/β-catenin in OL function that may largely depend on the timing, context, and cellular environment. Taken together, activation of WNT/β-catenin signaling in demyelination appears to inhibit OL development and decreases myelin production, resulting in a remyelination failure that could contribute to the development of the WML seen in cSVD.

## 6. Oligodendrocyte–Endothelial Cell Crosstalk

Although both ECs and OPCs/OLs are of importance for the BBB integrity, the interaction between ECs and OPCs/OLs and their role in cSVD remain largely unclear. It has been suggested that EC dysfunction might alter OL function, and vice versa [10]. There is no direct evidence linking EC–OL interaction and cSVD. However, early investigations on the SHRSP, a hypertensive rat model that recapitulates many brain structural abnormalities characteristic of cSVD pathology, including WMLs, suggest that endothelial dysfunction precedes and leads to alterations in myelin and white matter [73,74,75]. A reciprocal interaction between ECs and OLs was also demonstrated in an animal model of neurofibromatosis. OL-specific overactivation of Ras signaling in these animals led to an aberrant production of NO in addition to dysregulation of TJ proteins in both ECs and myelin sheaths. Ras overexpression also led to enlarged perivascular spaces and BBB leakages, which correlated with myelin breakdown. This indicates that OL-specific changes might have an effect on ECs and lead to features of cSVD pathology [76]. In addition, mice with endothelial dysfunction presented increased remnants of capillaries in the form of string vessels and decreased numbers of OPCs in the white matter [77]. These abnormalities predate other cSVD pathology in both animal models and human post-mortem tissue, suggesting an interplay between the endothelium and myelinating cells [77,78]. 

Aria and Lo were the first to describe a clear interaction between ECs and OLs in an in vitro model by culturing rat OPCs in human brain EC-conditioned medium, which induced an increased OPC proliferation rate. They suggested that EC-secreted factors, such as fibroblast growth factor (FGF) and BDNF, mediated these effects [79]. In turn, OPCs seem to affect ECs in culture as well. Culturing ECs in conditioned medium from OPCs led to EC proliferation and angiogenesis by secreted factors, such as transforming growth factor (TGF)-β and matrix metalloproteases (MMPs). OPCs support BBB integrity by releasing either TGF-β1 in normal conditions, or disrupt it and induce angiogenesis by releasing MMP-9 when stressed by inflammatory cytokines [80,81]. Taken together, these results indicate that factors released by ECs and OPCs play an important role in promoting their interactions and influencing their functions.

More recently, abnormal WNT signaling was suggested to alter the EC–OPC interaction, leading to abnormal OPC migration and dysfunction of both ECs and OLs [82,83]. Hence, we performed a systematic literature search to investigate the exact role of WNT signaling in the interaction between ECs and OPCs/OLs and its involvement in cSVD.

## 7. Literature Search Method

Publications on ECs and OPCs/OLs and interaction related to cSVD were exclusively identified through PubMed search engine and reported following the Preferred Reporting Items for Systematic review and Meta-Analysis Protocols (PRISMA-P) [84]. The search was conducted on 9 April 2020 and the following search terms and combinations were used to identify articles: Cerebral small vessel disease AND endothelial cells; Cerebral small vessel disease AND endothelium; Cerebral small vessel disease AND oligodendrocyte; Cerebral small vessel disease AND OPC; Cerebral small vessel disease AND WNT; Oligodendrocyte AND endothelial; OPC AND endothelial; Endothelial AND interaction AND (oligodendrocyte OR OPC OR OPC); Endothelial AND oligodendrocyte AND WNT; Endothelial AND OPC AND WNT; WNT AND angiogenesis; WNT AND BBB AND oligodendrocyte; WNT AND hypoperfusion; WNT AND hypertension; WNT7 AND endothelial; WNT7 AND oligodendrocyte. Two additional relevant papers were later added by the authors.

**Figure 2:**
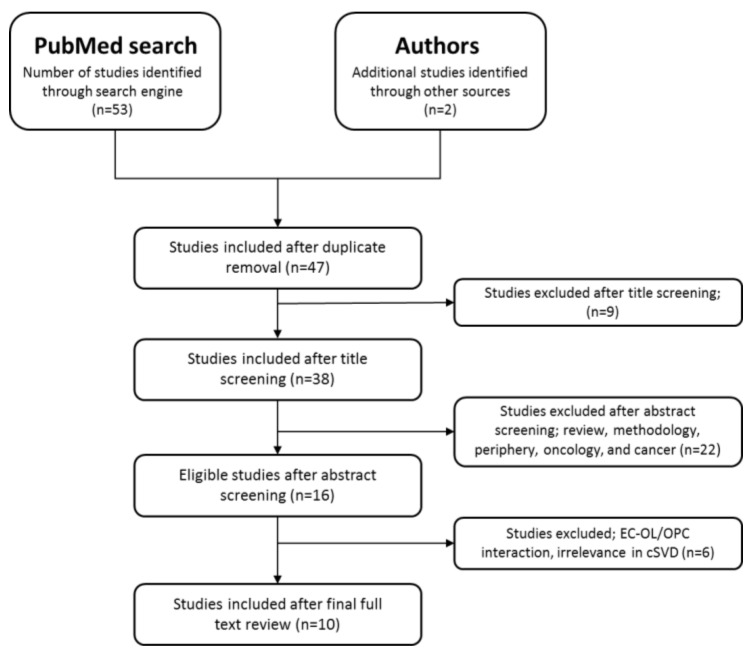
Flow diagram showing the systematic Preferred Reporting Items for Systematic review and Meta-Analysis Protocols (PRISMA-P) search protocol and identification, screening, and extraction of the corresponding studies. ECs, endothelial cells; OLs, oligodendrocytes; OPCs, oligodendrocyte precursor cells; cSVD, cerebral small vessel disease.

Duplicate papers were removed, and studies underwent an initial screen based on their title, followed by a screening of their abstract. Studies using the following terms were included during title screening: Endothelial cells; oligodendrocytes and/or oligodendrocyte precursor cells; and cerebral small vessel disease. Papers using the following terms were included after screening the abstract: Interaction EC–OPC/OL; inflammation; vasculature; cerebrovascular; animal and human studies. We excluded publications matching the following topics: Review, methodology, periphery, oncology, and cancer. Furthermore, studies were excluded if they referred to EC interaction with OPCs or OLs in the context that was not related to the brain or irrelevant for cSVD (e.g., WNT signaling in brain tumor environment) after full text reviewing. There were no commentaries, editorials, rectifications, or non-English publications in our search results. Screening and extraction of articles was performed by the first author (NM) under the guidance of the other authors. For each study, the following variables were recorded: Language, year of publication, type of disease, clinical design, experimental design, animal model, and results. The results are described in a flow diagram (Figure 2).

## 8. WNT Signaling in Endothelial Cell–Oligodendrocyte Crosstalk

The key role of WNT/β-catenin signaling in various brain cells is subject to complex regulation. Miyamoto et al. reviewed recent findings indicating a WNT-dependent single cell migration of adult OPCs from the subventricular zone to damaged areas under the guidance of ECs, which may contribute to white matter recovery [83]. Other findings supporting a key role for WNT signaling in white matter recovery via OPC recruitment include the initial upregulation of WNT expression in OPCs during hypoxia, WNT-mediated migration of OPCs, and an early increase of OPCs after injury [50,52,85,86]. Evidence for a reciprocal interaction came from Iijima et al. by the induction of remyelination due to EC dysfunction via endothelin (ET)-1 injection, indicated by a decrease in immunohistochemical neuron-glial antigen 2 (NG2) and myelin basic protein (MBP) levels, which was rescued by transplantation of healthy ECs. In addition, the results suggested a potential intrinsic rescue mechanism via an increase in OPC density, 7 days after ET-1 injection [87]. EC dysfunction, indicated by an impaired dilatation response to acetylcholine (ACh) and calcitonin gene-related peptide (CGRP), and hypoperfusion, marked by an increased number of string vessels, due to TGF-β1 overexpression in ECs in a transgenic mouse model led to a decreased OPC density in white matter areas and cognitive impairment [77]. Treatment of EC dysfunction with simvastatin, a drug that improves overall endothelial function [88], counteracted these negative effects, possibly via mitogen-activated protein kinase/extracellular signal-regulated kinase (MAPK/ERK) signaling [77]. This might indicate a cross-talk with the WNT/β-catenin signaling pathway in the interaction of EC–OL, as MAPK can increase β-catenin expression through direct phosphorylation of GSK3β by p38 [89]. These indirect evidences suggest that EC dysfunction precedes OL dysfunction and white matter abnormalities, possibly via WNT signaling. We thus suggest that WNT/β-catenin signaling modulates both EC and OL function and their interaction with potential pleiotropic roles in cSVD. 

In cSVD animal models and patients, increased OPC proliferation and migration has been observed following EC dysfunction [78,90]. Rajani et al. showed that SHRSP rats and human post-mortem tissue displayed EC abnormalities before any classical signs of cSVD pathology or stroke. Direct evidence for the ECs and OPCs/OLs interaction can be found in in vitro assays with OPCs cultured with conditioned media from SHRSP rat primary brain microvascular EC cultures, which exhibit decreased OPC differentiation and increased proliferation [78]. In vitro and in vivo increased OPC density and proliferation together with an initial increase in OPCs, as observed in patients with leukoencephalopathy, suggests an attempt by the brain to recruit myelinating cells for white matter repair [78,87,90]. In fact, WNT signaling is involved in the migration of OPCs using the vascular tree [72,85,86]. OPCs respond to demyelination by becoming activated, proliferating, migrating, and ultimately differentiating into myelinating OLs [72]. Under physiological conditions, demyelination results in the activation of WNT signaling in OPCs, which in turn leads to the upregulation of C-X-C chemokine receptor type 4 (Cxcr4). This protein binds to its ligand stromal cell-derived factor 1 (Sdf1, also known as C-X-C motif chemokine 12 [Cxcl12]) expressed by ECs and mediates the single cell migration of OPCs to the recruitment site [85,86]. Tsai et al. showed that overexpressing Cxcr4 led to increased attachment of OPCs to the vasculature and OPC clustering, which could be reversed by inhibiting the Cxc4–Sdf1 interaction. Once these OPCs are at their intended destination, OPCs can detach from the vessel and differentiate into mature myelinating OLs due to the downregulation of WNT and Cxcr4. However, in disease states, abnormal EC function was shown to result in disruption of OPC migration, OPC clustering, delayed OPC differentiation, and a decrease in myelination. These effects might involve WNT signaling pathways as upregulation of Cxcr4 was detected in WNT-activated OPC clusters [85]. OPC clusters were shown to be present in lesions in both animal models and human white matter injury, where aberrant OPC migration and remyelination seem to be associated with overactive WNT signaling [86]. These effects are both autocrine and paracrine, thus also affecting surrounding ECs. OPCs are indeed able to activate WNT-mediated angiogenesis [52,72], and regulate it via the expression of c-Cbl, acting as a negative feedback mechanism for WNT/β-catenin signaling [58]. These results indicate an important role for OPC clustering in the dysregulation of EC and OPC function.

OPC clustering, and the dysfunctional overexpression of WNT signaling seen in Olig2-cre: APC floxed/floxed mice, in turn lead to EC dysfunction, BBB disruption, and inflammation in non-injury settings [86]. A key component in this process is the WNT-mediated expression of WNT inhibitory factor 1 (Wif1), which is expressed following β-catenin activation. Wif1 is highly upregulated in OPCs, activated due to WNT/β-catenin signaling, and functions as a negative feedback control mechanism to decrease WNT activation in OPCs. However, overexpression of Wif1 also has a paracrine effect on ECs by downregulating the TJ protein claudin5, leading to BBB dysfunction and subsequent neuroinflammation [86]. Altogether, WNT/β-catenin signaling and its target genes are involved in the tight regulation of the BBB and angiogenesis to adjust the blood supply according to demand. However, this is a complex synergistic interaction since oxygen levels also influence WNT expression [52].

Hypoxic conditions lead to the upregulation of WNT7a and WNT7b, mediated by the stabilization of hypoxia-inducible factor (HIF)1/2α, which resulted in decreased myelination in mouse white matter [52,91]. These effects were normalized by XAV939, an inhibitor of WNT/β-catenin-mediated transcription, via the stabilization of Axin [92]. Cell culture experiments also demonstrated an arrest of OPC maturation when WNT signaling was overactive. In vitro, OPC-conditioned media promoted β-catenin-mediated endothelial tip sprouting and tube formation in mouse brain ECs, which was inhibited by XAV939 [52]. The secretion and release of WNT7a and WNT7b proteins from OPCs was shown to directly induce angiogenesis and this was prevented by XAV939, demonstrating that this effect was WNT/β-catenin dependent [52]. Wang et al. suggested that OPCs might have a positive effect in reducing the infarct volume and brain edema and improving cognitive function after middle cerebral artery occlusion in mice [82]. Transplantation of OPCs into the infarct area reduced protein leakage into the brain parenchyma, and rescued claudin5 expression. This was associated with an upregulation of endothelial β-catenin and inhibition of WNT/β-catenin signaling, as using XAV939 increased BBB leakages and decreased claudin5 expression. These results are in agreement with in vitro findings showing a decreased permeability of brain ECs treated with either WNT7a or OPC-conditioned medium. In addition, WNT7a was upregulated in mice transplanted with OPCs, suggesting a key role in this interaction. OPC-conditioned media or WNT7a treatment increased β-catenin and claudin5 expressions in brain ECs, an effect that was reversed by WNT7a knockdown in cultured OPCs [82]. Interestingly, recent evidence suggests that M2c anti-inflammatory microglia secrete WNT7a and promote oligodendrogenesis, indicating that pleiotropic actions are also involved in microglia and OL crosstalk [93].

## 9. Discussion

**Figure 3:**
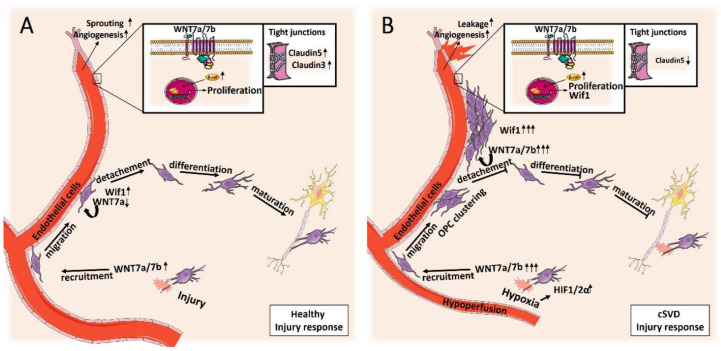
Schematic overview of the proposed contribution of WNT/β-catenin signaling in cSVD. (**A**) In a healthy situation, hypoxic injury induces WNT7a and WNT7b signaling in OPCs/OLs to promote the recruitment of new OPCs at sites of demyelination. OPCs migrate to the site of injury partly by using the physical support offered by cerebral vessels. WNT/β-catenin signaling in ECs promotes angiogenesis to supply oxygen and necessary nutrients for recovery. Once OPCs have reached their destination, feedback mechanisms are activated via Wif1, leading to the downregulation of WNT/β-catenin signaling and detachment from cerebral vessels. Subsequently, angiogenesis stops and OPCs differentiate into mature OLs, capable of initiating remyelination. (**B**) In cSVD, activation of HIF1/2α due to local hypoxia results in an exaggerated upregulation of WNT7a and WNT7b, which is aggravated by EC dysfunction. This leads to dysfunctional OPC migration and clustering. Local angiogenesis induced by WNT/β-catenin signaling results in a leaky and inflamed BBB partly due to the expression of negative feedback proteins that alter the expression of the tight junction protein claudin5. In addition, decreased detachment and differentiation of OPCs in turn leads to attenuated remyelination and white matter repair.

In summary, direct and indirect evidence suggests an interplay between ECs and OPCs/OLs driven by WNT/β-catenin signaling. The mechanistic/molecular hypothesis of their interaction may differ between physiological and pathological conditions as proposed below (Figure 3). We suggest that the activation of WNT7a and WNT7b signaling in healthy ECs and OPCs/OLs in response to injury may help the recruitment of new OPCs at sites of demyelination via the structural support of cerebral vessels. In the meantime, this endothelial signaling promotes angiogenesis to supply oxygen and necessary nutrients for recovery. Once OPCs have reached their destination, feedback mechanisms are activated via Wif1, leading to the downregulation of WNT/β-catenin signaling. Subsequently, angiogenesis stops and OPCs detach from the vessels to differentiate into mature myelinating OLs. In cSVD however, local hypoxic injuries can alter the brain endothelium [52], causing activation of HIF1/2α, which in turn excessively upregulates WNT7a and WNT7b signaling. Dysfunctional ECs exhibit an overexpression of WNT proteins, leading to dysfunctional OPC migration and clustering, and local angiogenesis. The expression of negative feedback proteins can have paracrine effects, resulting in inflammation, abnormal angiogenesis, and a leaky BBB due to a low expression of the TJ protein claudin5 in inflamed or newly formed vessels. EC dysfunction and WNT signaling may also lead to decreased OPC detachment, leading to decreased OPC differentiation and decreased myelination. Ultimately, these effects may stimulate further damage to the brain by decreasing the rate of myelin repair, potentiating the development of WML and ensuing cognitive impairment.

## 10. Conclusions

It this review, we identified an important role for WNT signaling in the EC–OPC/OL interplay, whose contribution (both autocrine and paracrine) should be further investigated. The use of animal and post-mortem human material will be determinative to decipher the pleiotropic role of WNT in this interaction and its relevance for cSVD pathobiology and other cerebrovascular disorders. Beyond a greater pathological understanding, it may bring therapeutic opportunities to seal an impaired BBB, reverse OPC clustering, and repair white matter injuries in cSVD.
